# Genetic characterization and phylogeny of pigeon paramyxovirus isolate (PPMV-1) from Pakistan

**DOI:** 10.1186/s40064-016-2939-1

**Published:** 2016-08-08

**Authors:** Sameera Akhtar, Muhammad Akram Muneer, Khushi Muhammad, Muhammad Yasin Tipu, Masood Rabbani, Aziz ul-Rahman, Muhammad Zubair Shabbir

**Affiliations:** 1 Department of Microbiology, University of Veterinary and Animal Sciences, Lahore, 54600 Pakistan; 2 Department of Pathology, University of Veterinary and Animal Sciences, Lahore, 54600 Pakistan; 3 Quality Operations Laboratory, University of Veterinary and Animal Sciences, Lahore, 54600 Pakistan

**Keywords:** Pigeon paramyxovirus type 1 (PPMV-1), Newcastle disease virus, Pigeon, Pakistan

## Abstract

**Background:**

Knowing the genome characteristics of circulating Newcastle disease viruses [avian paramyxoviruses (APMV-1) and pigeon paramyxoviruses (PPMV-1)] is important to devise appropriate diagnostics and control strategies. APMVs originating from chicken and wildlife in Pakistan are well-elucidated; nevertheless, molecular characterization for the circulating PPMV-1 is largely unknown.

**Findings:**

Here, we have performed fusion (F) and hemagglutinin (HN) gene based characterization of PPMV-1 isolated from an outbreak in a pigeon flock. With F_0_ proteolytic cleavage site (_112_RRQKR↓F_117_), characteristic of velogenic/mesogenic serotype, the complete F and HN gene based sequence analysis of the isolate revealed evolutionary relationship to genotype VI. Further analysis of hyper-variable region of F-gene demonstrated clustering of the study isolate with genotype VIb. The deduced residue analysis for both F and HN protein showed a number of substitution mutations in the functional domains distinct from representative strains of each genotype including the vaccine strains; some of them were found exclusive to the study isolate.

**Conclusions:**

Though limited and preliminary data, the findings enhance our knowledge towards circulating strains of PPMVs in Pakistan. Further studies are needed to ascertain its potential for transmission in the wild birds, commercial and backyard poultry and its subsequent shedding into the environment.

## Background

Pigeon Paramyxovirus type 1 (PPMV-1) is an antigenic and host variant of classical Newcastle Disease virus (NDV) or Avian Paramyxovirus type 1 (APMV-1) that causes Newcastle Disease (ND)-like infection and pathology in pigeons (Ujvari et al. 2003). Including the earliest known PPMV-1 (Iraq78) originating from the Middle-East (ME), and the strains responsible for third panzootic in 80 s involving a pigeon-adopted APMV-1 are considered likely to be derived from these ME viruses (Kaleta et al. 1985; Ujvari et al. 2003) and as yet continue to circulate around the globe. The virus has an approximate genomic length of 15192 nt and, despite characteristic F protein cleavage site for velogenic strains (_112_GRQKRF_117_ or _112_RRKKRF_117_ or _112_RRQKRF_117)_, differences in pathogenicity index range from moderate to no virulence for chicken (Collins et al. 1994; Dortmans et al. 2010). However in recent years, some PPMV-1 have been reported to be highly pathogenic for chicken after passage either in chicken or chicken embryo indicating their potential to cause ND outbreaks (Dortmans et al. 2011). Today, ND caused by either virulent APMV-1 or PPMV-1 is considered endemic to feral and domestic pigeon (Columbiformes) worldwide (Aldous et al. 2004). Clinical symptoms differ depending upon host immune titre and virulence of isolate involved (Dortmans et al. 2011). Most of the times, the observed clinical symptoms relate to neurotropic form of ND including tremors, torticollis and disturbed equilibrium, however naturally infected pigeon also exhibit respiratory symptoms such as sneezing, coughing and tracheal rales (Marlier and Vindevogel 2006).

Since the establishment of well-organized poultry sector in Pakistan to-date, there have been a number of epidemics of ND in commercial poultry, wild birds and domestic pigeons. Although, APMV-1 from commercial poultry and wild birds have been isolated and well-characterized as genotype VI and VII (Munir et al. 2012; Shabbir et al. 2012, 2013a, b), despite a number of clinically suspected outbreaks in pigeons including vaccinated ones (Lab data, not published), there is absolute paucity of information pertaining to circulating lineage of PPMV-1 and its nucleotide and amino acid profile to reference and vaccinal strains. We analysed complete F and HN genes of an isolate recovered from ND outbreak in a pigeon flock. The obtained sequences were processed for phylogeny and amino acid residue analyses giving an insight towards genetic diversity of the indigenous strain and its comparative evolution to those reported earlier.

## Methods

A Newcastle disease outbreak occurred in a flock of racing pigeons (n = 56) in district Lahore, Pakistan (31.5790°N, 74.3096°E) during December, 2014. Four days post appearance of clinical symptoms, 13 died and 29 were morbid. The clinical symptoms observed in affected pigeons were circling movement and tremors while some birds were also exhibiting mild respiratory symptoms such as sneezing, coughing and nasal discharge. Necropsy was performed and samples (trachea, lungs, and brain) were processed for isolation of NDV in 9 day old embryonated chicken eggs (ECE). Presence of agglutinating virus was confirmed in harvested allantoic fluid by spot agglutination assay using 10 % chicken red blood cells (RBCs). Later, identity of the isolate was confirmed as NDV through standard haemagglutination inhibition assay using specific antisera.

Total RNA was extracted from allantoic fluid using commercially available RNA extraction kit as per manufacturer’s instructions (QIAamp Viral RNA Mini Kit, Qiagen, USA). Quantity (NanoDrop, USA) and quality (QubitFlourometer, USA) of extracted RNA was measured. The extracted RNA was subjected to amplification of complete F and HN genes spanning the genomic region from 4498 to 6330 nucleotide through reverse transcription polymerase chain reaction (RT-PCR) using the primers and protocols as described previously (Munir et al. 2010). The amplified products were purified by 1.0 % gel electrophoresis, using the Wizard^®^ SV Gel and PCR Clean-Up System (Promega, Co., Madison, WI, USA) as per manufacturer’s instructions. Using the same primers (as used previously for F and HN gene amplification) and ABI PRISM BigDye Terminator version 3.1 (Applied Biosystems, Foster City, CA, USA), the purified genomic DNAs were processed for sequencing reaction on a 3100 DNA Analyzer (Applied Biosystems, Foster city, CA, USA). Each genomic fragment was sequenced twice in both forward and reverse directions to generate a reliable consensus sequence. The consensus sequences of F and HN genes of the study isolate (Pi/MZS1-UVAS/2014) has been submitted to GenBank under the accession number KU644586 and KU644587, respectively.

The obtained sequences and sequences reported earlier (GenBank) were aligned in BioEdit version 5.0.6 (Hall, 1999) using ClustalW and cut to equal lengths. Phylogenetic relationships of complete F, HN gene and hyper-variable region of F gene of study isolate were elucidated to the corresponding region of previously characterized viruses around the globe (http://www.ncbi.nlm.nih.gov/nuccore/?term=partial+F+gene+of+Newcastle+disease+virus) at the level of genotype and sub-genotype using the MEGA version 6.0 software (Tamura et al. 2013). The evolutionary distances were inferred and expressed based on the number of nucleotide substitutions per site. The codon positions included in the analysis were the 1st, 2nd, 3rd, and noncoding. All positions containing gaps and missing data were eliminated from the data set (the “complete deletion” option). Furthermore, comparative residue analysis of the representative strains of each known genotype was analysed through BioEdit.

## Results and discussion

**Fig. 1:**
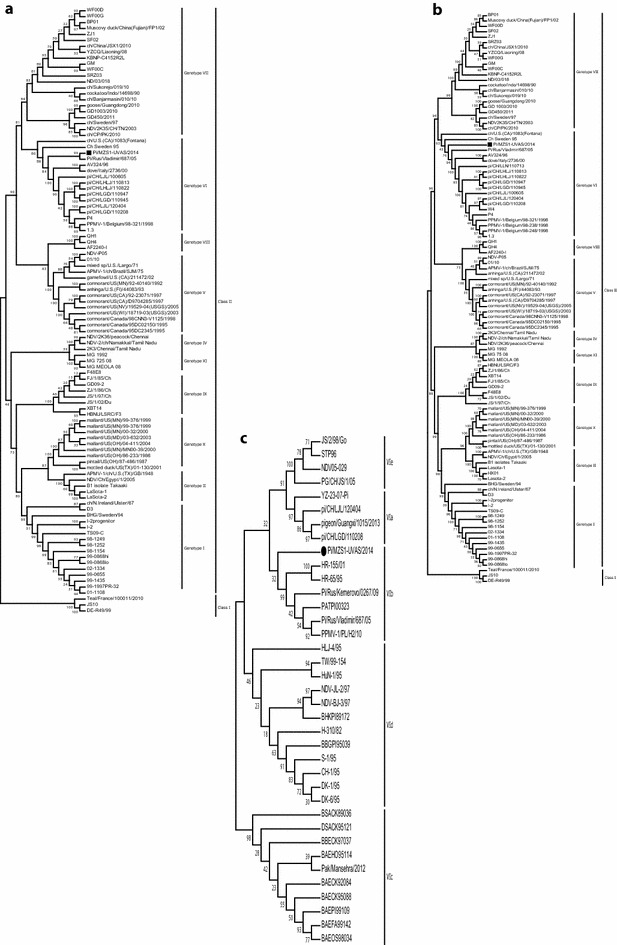
Phylogenetic consensus tree for the pigeon-originated NDV isolate for fusion gene (**a**), hemagglutinin gene (**b**) and hypervariable region of F gene (**c**). The nucleotide sequences of study isolate for each gene were compared with corresponding genes of representative strains reported previously to public database, the GenBank. The evolutionary history was inferred using the Neighbor-Joining method with 1000 bootstrap value in MEGA version 6.0

Agglutination of harvested virus from allantoic fluid with chicken RBCs (1:128) and subsequent inhibition with specific antisera (1:128) confirmed the presence of NDV. Since a particular emphasis is given to F and HN gene-based molecular characterization of NDV strains in the previous studies (Toyoda et al. 1988; Yussof and Tan, 2001; Aldous et al. 2003; Ujvari et al. 2003; Kim et al. 2008; Munir et al. 2012; Shabbir et al. 2012), we also used complete F and HN gene as well as hyper-variable region of F-gene (47–421 nt, 375 bp) to determine phylogenic relationship of the study isolate to previously known representative strains of APMV-1 and PPMV-1 around the globe at the level of genotype and sub-genotype. Based upon complete F and HN gene phylogeny, the study isolate clustered within genotype VI close to an isolate reported previously from Russia (Pi/Rus/Vladimir/687/05) (Fig. 1a, b). Further analysis of hyper-variable region of F gene revealed clustering of the study isolate to sub-genotype VIb (Fig. 1c). The clustering of study isolate close to the ones from Russia highlights potential ancestor or origin of the virus that may involve migratory route of birds from North to South. Since the first introduction of genotype VI, various strains such as VIa, VIb, VIc and VIdresponsible for pigeon-origin panzootics have been reported from time to time with widespread geographic movement (Kaleta et al. 1985, Ujvari et al. 2003; Aldous et al. 2004; Lee et al. 2004). The most recent example is the identification of an isolate of genetic clade VIa from China (pi/GX/1015/13) that is supposed to have ancestor similar to isolate (W4/2005) from Europe (Wang et al. 2015). It is noteworthy that NDVs belonging to genotype VI was identified from chicken in district Karachi back in 2005 (Khan et al. 2010) and, more recently, genotype VIc was reported from a commercial poultry farm in northern areas of Pakistan (Shabbir et al. 2013a). As the presence of cleavage motif in mesogenic strains does not necessarily correlate with the pathogenicity for chickens (Kommers et al. 2002; Ujvari et al. 2003; Kim et al. 2008), studies ascertaining transmission of pigeon-originated NDVs in vaccinated as well as immunologically naïve flocks and their subsequent shedding are essential in understanding their potential to cause disease in wild, commercial and backyard poultry in Pakistan.

**Fig. 2:**
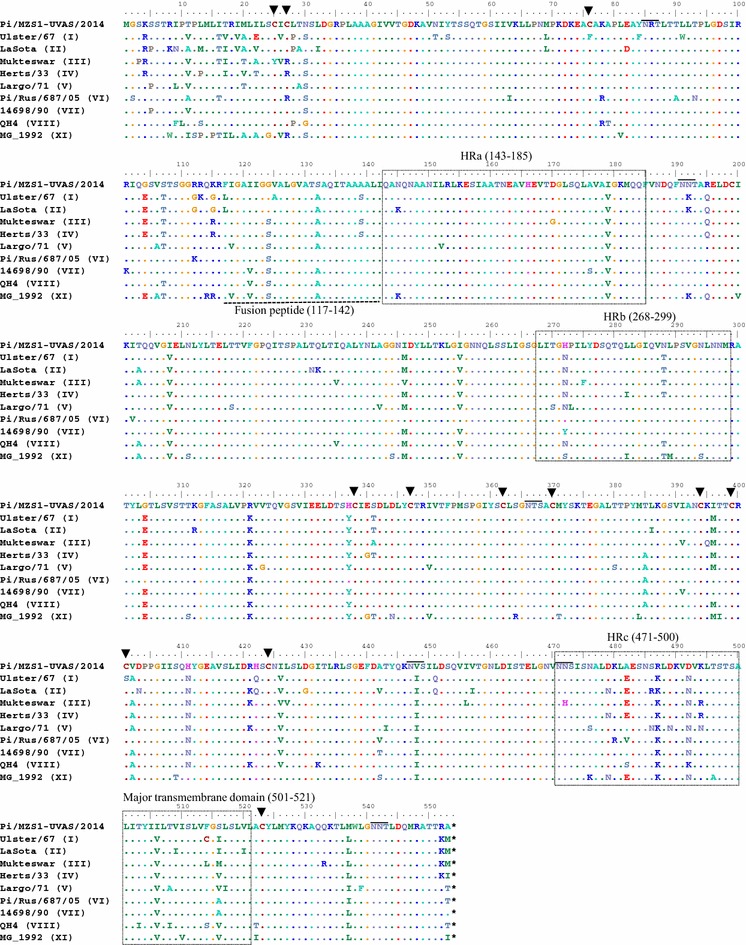
Alignment of deduced amino acid sequence of complete F gene of pigeon isolate. The residue profile of study isolate is compared with strains of NDVs representing different genotypes including the vaccine strains. Structurally and functionally important residues are *boxed* and *highlighted*

Nucleotide and amino acid residues of the isolate were identified in the complete coding region of the F (4550–6211, 1662 bp, 553 aa) and HN (6418–8133, 1716 bp, 571 aa) genes. Although, differences in amino acid length of HN protein have been reported previously from different strains of NDV (Romer-Oberdorfer et al. 2006), the study isolates had open reading frame that encoded 571 residues, a feature common to most of the virulent NDVs (Tirumurugaan et al. 2011). While comparing nucleotide and residues of the study isolate to representative strains of different genotypes, the percent similarity for F gene was found to be maximum with genotype VI (92.4 and 94.2) while it was observed to vary from 84 to 90 for genotype representing vaccine strains; it was 84.2 and 88.2 for genotype II and 85.8 and 90.2 for genotype III. Similarly for HN gene, the percent similarity for genotype II, III and VI was found to be 82.3 and 87.9, 85.5 and 89.8, and 92.1 and 93.6, respectively. Within hyper-variable region of the F-gene (47–421 nt), we observed predicted multiple basic amino acid residues at proteolytic cleavage site (F0) considered typical of mesogenic or velogenic strains (Aldous et al. 2003). The corresponding residues at the F2 protein and the N-terminus of F1 protein were found to be _112_RRQKR_116_ and phenylalanine (_116_↓F_117_), respectively. This is similar to PPMVs reported after 80 s as the cleavage motif for isolates before 80 s was usually_112_GRQKR↓F_116_ (Meulemans et al. 2002). Six potential glycosylation sites [Asp(N)-X-Ser(S)/Thr(T), X could be any residue except proline (P) and aspartic acid (D)] including _85_NRT_87_, _191_NNT_193_, _366_NTS_368_, _447_NVS_449_, _471_NNS_473_, _541_NNT_543_ were identified in the F protein. Although there were variations in residue composition of glycosylation sites for different genotypes, the 3rd glycosylation site was found to be unique in a way that all the representative strains of each genotype had residues (_447_NIS_449_) in comparison to the study isolate; Val (V) was present at position 448. Moreover, 13 cysteine (C) residues located at position 25, 27, 76, 199, 338, 347, 362, 370, 394, 399, 401, 424 and 523 were observed in the study isolate. The sites for glycosylation and cysteine residues are thought to be conserved (Umali et al. 2013), however, we found differences in residues composition for a given glycosylation site and variations in both number and site of cysteine residues. For example, there were differences in composition of residues in glycosylation sites located at position 191–193, 447–449 and 471–473. Likewise, compared to the study isolate, we found 12 cysteine residues in Ulster-67 (genotype-I) with replacement of two residues [76(C → F) and 401 (C → S)] and addition of one cysteine residue at position 514 than the study isolate (G). While characterizing pigeon isolate (PPMV-1) from China, Wang et al. (2015) reported 13 cysteine residues in one of six isolates in a pattern similar to the study isolate with additional residue (13th) at position 27. Compared to consensus sequence of vaccine strains used in Pakistan and other representative strains, we observed a number of substitution mutations in residue sequences for fusion peptide (117–142 aa) at position 121 (V → I), 124 (S → G) and 132 (A → S), hydrophilic region-a (143–185 aa) at position 179 (V → I), hydrophilic region-b (268–299 aa) at position 272 (N → H), 288 (T → N) and hydrophilic region-c (471–500 aa) at position 482 (E → A), 487 (K → R), 492 (N → D). Mutation at position 506 (V → I) and 516 (I/M → S) of the major transmembrane region (501–521 aa) further indicates lack of conserveness of this particular part of genome (Fig. 2).

**Fig. 3:**
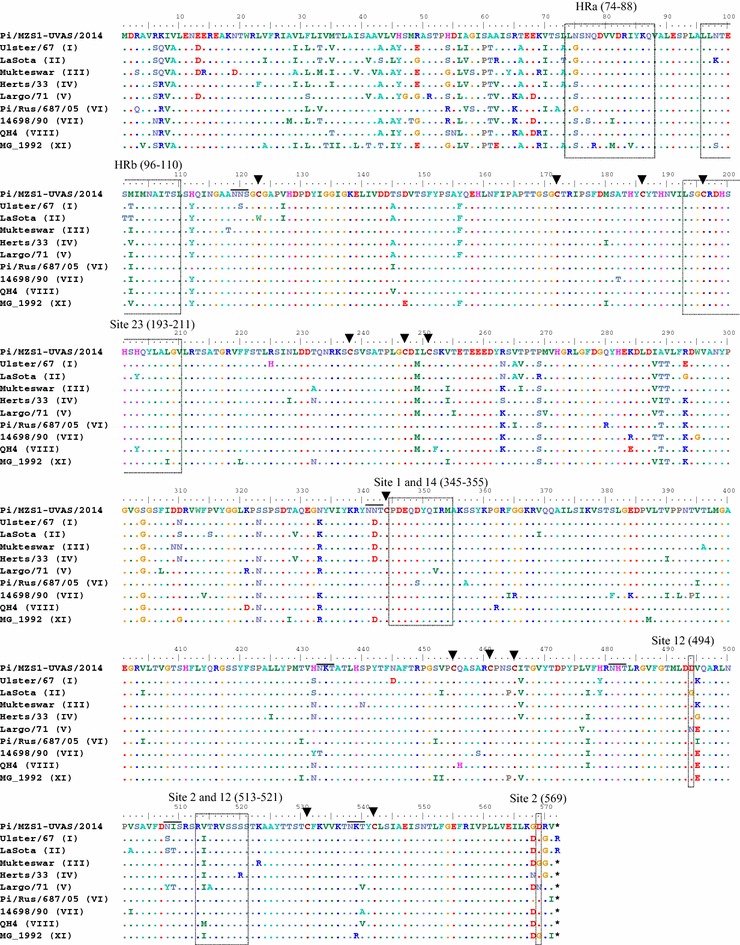
Alignment of deduced amino acid sequence of complete HN gene of pigeon isolate. The residue profile of study isolate is compared with strains of NDVs representing different genotypes including the vaccine strains. Structurally and functionally important residues are *boxed* and *highlighted*

On the other hand, for HN gene, analysis of predicted amino acid sequence revealed conserveness of cysteine residues at positions 123, 172, 186, 196, 238, 247, 251, 344, 455, 461, 465, 531 and 542; key residues for receptor (sialic acid) binding at positions R174, E401, R416, Y526; residues for the hydrophobic core of the stalk 4HB at positions Y85, V88, S92, L96, T99, I103, I107, L110 and I114; and stalk residues involved in direct interaction with F protein at positions R83, A89, L90, L94 and L97 (Ke et al. 2010; Yuan et al. 2011). Six N-glycosylation sites at positions _119_NNS_121_, _341_NNT_343_, _433_NKT_435_, _481_NHT_483_, _508_NIS_510_, and _538_NKT_540_ were identified. When compared to most common vaccine strain used in Pakistan (genotype II), beside substitutions at several point in total length of HN gene, we found substitutions in the transmembrane domain (I29V, T33I, V35M, S43V, L45V, Y46H, G49R), antigenic sites (K98N, G494D, I514V) and neutralization epitope (N263R) (Fig. 3). It is interesting to note that some substitutions were found to be exclusive to the study isolate notably at positions V179I, K321R, I448V, K487R, K/N492D, V506I, L537M for F protein and Q/R7K, V8I, T61A, S/G75N, S/R269P, I/T289A for HN protein (Figs. 2, 3). This is important as some geographically conserved mutation have been reported recently; K4I is conserved for strains reported from Japan (Umali et al. 2013) while I52V, K78R, R101K are considered conserved for isolates originating from the Far East Asia (Japan, China and Taiwan). Taken together, variation in nucleotide and subsequent substitutions/alternations in amino acid profile such as observed in this study is consistent with previously described theories of evolution of RNA viruses particularly NDV (Yu et al. 2001; Umali et al. 2013).

As the pigeon flock had a history of vaccination with lentogenic strain (LaSota), identification of cleavage motif similar to velogenic raises concerns for the type of vaccine used to vaccinate the flock and the need for post-vaccine evaluation. Substitution and subsequent mutations at fusion peptide, HR regions, trans-membrane domain and antigen neutralization sites could affect the fusion activity of NDVand, alteration in antigenic epitopes particularly those that are involved in virus attachment, could result in escape variants and subsequent vaccine failure (Cho et al. 2007; Umali et al. 2014; Wang et al. 2015). Beside potential compromise in procedures used in vaccine storage and administration, the expected genetic distance between vaccine strains and the study isolate seems to be well-explained by Wang et al. (2015) through cross-HI assay. While evaluating the antigenic diversity of different strains through cross-HI assay, they reported a lower R-value (0.13–0.18) for interaction of PPMVs to LaSota than between PPMVs (VIa and VIb, 0.7) indicating an obvious antigenic difference with vaccine strain.

## Conclusion

We characterized the circulating genotype of pigeon (PPMV-1) closely related to previously known clades of VIb. Since outbreaks of NDV in commercial poultry has been reported in one of the province of Pakistan from a closely related genotype (VIc) to the study isolate (VIb), future studies relating to disease surveillance coupled with experiments involving potential to transmit and shedding from either vaccinates or non-vaccinates are essential. Moreover, immunity barrier provided by LaSota and other vaccinal strains needs to be evaluated in the face of newly emerging NDV strains.

## References

[CR1] Aldous EW, Mynn JK, Banks J, Alexander DJ (2003). A molecular epidemiological study of avian paramyxovirus type 1 (Newcastle disease virus) isolates by phylogenetic analysis of a partial nucleotide sequence of the fusion protein gene. Avian Pathol.

[CR2] Aldous EW, Fuller CM, Mynn JK, Alexander DJ (2004). A molecular epidemiological investigation of isolates of the variant avian paramyxovirus type 1 virus (PPMV-1) responsible for the 1978 to present panzootic in pigeons. Avian Pathol.

[CR3] Cho SH, Kim SJ, Kwon HJ (2007). Genomic sequence of an antigenic variant Newcastle disease virus isolated in Korea. Virus Genes.

[CR4] Collins MS, Strong I, Alexander DJ (1994). Evaluation of the molecular basis of pathogenicity of the variant Newcastle disease viruses termed “pigeon PMV-1 viruses”. Arch Virol.

[CR5] Dortmans JC, Fuller CM, Aldous EW, Rottier PJ, Peeters BP (2010). Two genetically closely related pigeon paramyxovirus type 1 (PPMV-1) variants with identical velogenic fusion protein cleavage sites but with strongly contrasting virulence. Vet Microbiol.

[CR6] Dortmans JC, Koch G, Rottier PJ, Peeters BP (2011). A comparative infection study of pigeon and avian paramyxovirus type 1 viruses in pigeons: evaluation of clinical signs, virus shedding and seroconversion. Avian Pathol.

[CR7] Hall TA (1999). BioEdit: a user-friendly biological sequence alignment editor and analysis program for windows 95/98/NT. Nucl Acid Symp Ser.

[CR8] Kaleta EF, Alexander DJ, Russell PH (1985). The first isolation of the avian PMV-1 virus responsible for the current panzootic in pigeons?. Avian Pathol.

[CR9] Ke GM, Chuang KP, Chang CD, Lin MY, Liu HJ (2010). Analysis of sequence and haemagglutinin activity of the HN glycoprotein of New-castle disease virus. Avian Pathol.

[CR10] Khan TA, Rue CA, Rehmani SF, Ahmed A, Wasilenko JL, Miller PJ, Afonso CL (2010). Phylogenetic and biological characterization of Newcastle disease virus isolates from Pakistan. J Clin Microbiol.

[CR11] Kim LM, King DJ, Guzman H, Tesh RB, Travassos DA, Rossa APA, Bueno R, Dennet JA, Afonso CL (2008). Biological and phylogenetic characterization of pigeon paramyxovirus serotype 1 circulating in wild North American pigeons and doves. J Clin Microbiol.

[CR12] Kommers GD, King DJ, Seal BS, Carmichael KP, Brown CC (2002). Pathogenesis of six pigeon origin isolates of Newcastle diseases virus for domestic chickens. Vet Pathol.

[CR13] Lee YJ, Sung HW, Choi JG, Kim JH, Song CS (2004). Molecular epidemiology of Newcastle disease viruses isolated in South Korea using sequencing of the fusion protein cleavage site region and phylogenetic relationships. Avian Pathol.

[CR14] Marlier D, Vindevogel H (2006). Viral infections in pigeons. Vet J.

[CR15] Meulemans G, van den Berg TP, Decaesstecker M, Boschmans M (2002). Evolution of pigeon Newcastle disease virus strains. Avian Pathol.

[CR16] Munir M, Linde AM, Zohari S, Stahl K, Baule C, Holm K, Engstrom B, Berg M (2010). Complete genome analysis of an avian paramyxovirus type 1 strain isolated in 1994 from an asymptomatic black-headed gull (*Larus ridibundus*) in southern Sweden. Avian Dis.

[CR17] Munir M, Shabbir MZ, Yaqub T, Shabbir MA, Mukhtar N, Khan MR, Berg M (2012). Complete genome sequence of a velogenic neurotropic avian paramyxovirus 1 isolated from peacocks (*Pavo cristatus*) in a wildlife park in Pakistan. J Virol.

[CR18] Romer-Oberdorfer A, Veits J, Werner O, Mettenleiter TC (2006). Enhancement of pathogenicity of Newcastle disease virus by alteration of specific amino acid residues in the surface glycoproteins F and HN. Avian Dis.

[CR19] Shabbir MZ, Goraya MU, Abbas M, Yaqub T, Shabbir MAB, Ahmad A, Anees M, Munir M (2012). Complete genome sequencing of a velogenic viscerotropic avian paramyxovirus 1 isolated from pheasants (*Pucrasia macrolopha*) in Lahore, Pakistan. J Virol.

[CR20] Shabbir MZ, Abbas M, Yaqub T, Mukhtar N, Subhani A, Habib H, Sohail MU, Munir M (2013). Genetic analysis of Newcastle disease virus from Punjab, Pakistan. Virus Genes.

[CR21] Shabbir MZ, Zohari S, Yaqub T, Nazir J, Shabbir MAB, Mukhtar N, Shafee M, Sajid M, Anees M, Abbas M, Khan MT, Ali AA, Ghafoor A, Ahad A, Channa AA, Anjum AA, Hussain N, Ahmad A, Goraya MU, Iqbal Z, Khan SA, Aslam H, Zehra K, Sohail MU, Yaqub W, Ahmad N, Berg M, Munir M (2013a) Genetic diversity of Newcastle disease virus in Pakistan: a countrywide perspective. Virol J 10:170. http://www.virologyj.com/content/10/1/170 10.1186/1743-422X-10-170 PMC3681574 23721461

[CR22] Tamura K, Stecher G, Peterson D, Filipski A, Kumar S (2013). MEGA6: molecular evolutionary genetics analysis version 6.0. Mol Biol Evol.

[CR23] Tirumurugaan KG, Kapgate S, Vinupriya MK, Vijayarani K, Kumanan K, Elankumaran S (2011). Genotypic and pathotypic characterization of Newcastle disease viruses from India. PLoS One.

[CR24] Toyoda T, Gotoh B, Sakaguchi T, Kida H, Nagai Y (1988). Identification of amino acids relevant to three antigenic determinants on the fusion protein of Newcastle disease virus that are involved in fusion inhibition and neutralization. J Virol.

[CR25] Ujvari D, Wehmann E, Kaleta EF, Werner O, Savic V, Nagy E (2003). Phylogenetic analysis reveals extensive evolution of avian paramyxovirus type 1 strains of pigeons (*Columba livia*) and suggests multiple species transmission. Virus Res.

[CR26] Umali DV, Ito H, Suzuki T, Shirota K, Katoh H, Ito T (2013). Molecular epidemiology of Newcastle disease virus isolated from vaccinated commercial poultry farms in non-epidemic areas of Japan. Virol J.

[CR27] Umali DV, Ito H, Shirota K, Katoh H, Ito T (2014). Characterization of complete genome sequence of genotype VI and VII velogenic Newcastle disease virus from Japan. Virus Genes.

[CR28] Wang J, Liu H, Liu W, Zheng D, Zhao Y, Li Y, Wang Y, Ge S, Lv Y, Zuo Y, Yu S, Wang Z (2015). Genomic characterizations of six pigeon paramyxovirus type 1 viruses isolated from live bird markets in China during 2011 to 2013. PLoS One.

[CR29] Yu L, Wang Z, Jiang Y, Chang L, Kwang J (2001). Characterization of newly emerging Newcastle disease virus isolates from the People’s Republic of China and Taiwan. J Clin Microbiol.

[CR30] Yuan P, Swanson KA, Leser GP, Paterson RG, Lamb RA, Jardetzky TS (2011). Structure of the Newcastle disease virus hemagglutinin-neuraminidase (HN) extodomain reveals a four-helix bundle stalk. Proc Natl Acad Sci.

[CR31] Yussof K, Tan WS (2001). Newcastle disease virus: macromolecules and opportunities. Avian Pathol.

